# Butyrate suppresses expression of neuropilin I in colorectal cell lines through inhibition of Sp1 transactivation

**DOI:** 10.1186/1476-4598-9-276

**Published:** 2010-10-15

**Authors:** Danny CW Yu, Jennifer S Waby, Haridasan Chirakkal, Carolyn A Staton, Bernard M Corfe

**Affiliations:** 1 Department of Oncology, School of Medicine and Biomedical Sciences, University of Sheffield, Royal Hallamshire Hospital, Sheffield, S10 2JF, UK; 2 Current Address: Department of Biological Sciences, The University of Hull HU6 7RX UK

## Abstract

**Background:**

Neuropilin is a transmembrane receptor for vascular endothelial growth factor (VEGF) and is expressed in normal endothelial cells and upregulated in cancer cells. Neuropilin-1 (NRP-1) has been shown to promote tumour cell migration and survival in colon cancer in response to VEGF binding. The expression profiles of neuropilins, associated co-receptors and known ligands have been mapped in three colorectal cell lines: Caco-2, HCT116 & HT29. We have previously shown that butyrate, a naturally occurring histone deacetylase inhibitor (HDACi) produced by fermentation of fibre in the colon, causes apoptosis of colon cancer cell lines.

**Results:**

Here we demonstrate that butyrate down-regulates NRP-1 and VEGF at the mRNA and protein level in colorectal cancer cell lines. NRP-1 is a known transcriptional target of Sp1, whose activity is regulated by acetylation. NRP-1 down-regulation by butyrate was associated with decreased binding affinity of Sp1 for canonical Sp-binding sites in the NRP-1 promoter. siRNA-mediated knock-down of Sp1 implied that Sp1 may have strong DNA binding activity but weak transactivation potential.

**Conclusion:**

The downregulation of the key apoptotic and angiogenesis regulator NRP-1 by butyrate suggests a novel contributory mechanism to the chemopreventive effect of dietary fibre.

## Background

Fermentation of fibre in the colon leads to production of short-chain fatty acids (SCFA) including butyrate. Butyrate has been implicated in cellular homeostasis of the normal colonic mucosa, and this is thought to underwrite the chemoprotective effect of fibre [1]. *In vitro *studies indicate that butyrate causes cell cycle arrest, differentiation or apoptosis in a number of transformed cell lines. These outcomes are mediated by butyrate's inhibition of histone deacetylases (HDACs). Transcription factors Sp1 and Sp3 share canonical GC boxes and are thought to bind with equivalent affinity. Moreover, both Sp1 and Sp3 have been reported as acetylated and are targets for HDAC1 and HDAC2 [2,3]. Butyrate has been shown to inhibit HDAC activity thereby down-regulating Sp1 binding and up-regulating Sp3 binding. This leads to an increase in p21 expression, which ultimately causes cell cycle arrest [4]; and an increase in Bak expression which ultimately causes apoptosis [5]. Both events may contribute to the chemopreventive action of butyrate. In an accompanying paper, we use a novel anti-acetyl-Sp1 antibody to show that upregulation of p21, Bak and acetylation of Sp1 respond to the same subset of HDACi with highly similar EC50, implying a simple and causal relationship [6].

The vascular endothelial growth factor (VEGF) family comprises members that share structural homology and have been linked to cancer angiogenesis, metastasis and survival [7]. VEGF signalling on endothelial cells is mediated by three tyrosine kinase receptors VEGFR1-3 [8]. However, the Neuropilin (NRP) family of 130-140 kDa transmembrane glycoprotein receptors has recently been implicated in both VEGF-mediated angiogenesis [9] and colon cancer cell survival [10]. NRP-1 and NRP2 are non-tyrosine kinase receptors that bind with specific members of the VEGF family: NRP-1 binds VEGF_165_, VEGF-B, VEGF-E and placenta growth factor-2; whereas NRP-2 binds VEGF_145_, VEGF_165_, VEGF-C and VEGF-D [11]. NRP-1 is expressed in morphologically normal colonic epithelium [12] and is commonly over-expressed in human colon cancer where it correlates with advanced grade, metastatic potential [12], and decreased patient survival [13]. Cloning of the promoter region of NRP-1 demonstrated the presence of an AP-1, a CCAAT box and two Sp1 elements all of which contribute to induced promoter activity [14]. However, as yet it is not clear which of these elements is involved in the up-regulation of NRP-1 expression in colon cancer.

Patient data indicate that colorectal tumours with increased NRP-1 expression have a greater incidence of metastases, increased proliferation index and reduced numbers of apoptotic cancer cells than tumours with low NRP-1 staining [13] suggesting that NRP-1 may protect colon cancer cells from apoptosis. Interestingly siRNA-induced down-regulation of NRP-1 has been shown to increase sensitivity to chemotherapy by induction of apoptosis [15] suggesting that down-regulation of NRP-1 may have therapeutic potential. Therefore, modification of Sp family activity by butyrate and the potential of NRP-1 as an Sp1 target led us to investigate the ability of butyrate to modulate NRP-1 expression, with a view to providing an alternative therapy or chemopreventive strategy for colon cancer.

## Results

### Profile of angiogenic factors and their receptors mRNA expression in human colon cancer cell lines

**Figure 1:**
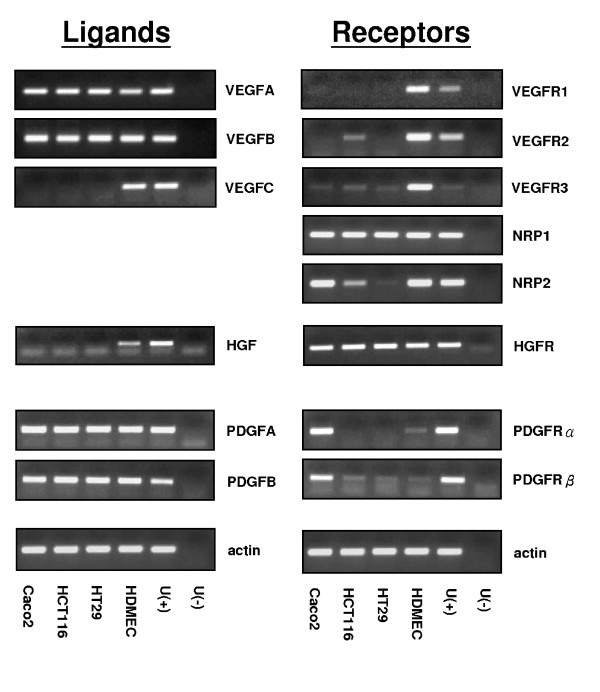
**mRNA expression of angiogenesis factors and their receptors in three human colon cancer cell lines**. VEGFA, VEGFB, VEGFC, VEGFR1, VEGFR2, VEGFR3, NRP-1, NRP2, PDGFA, PDGFB, PDGFRα, PDGFRβ, HGF and HGFR mRNA were extracted using Trizol and expression was determined by RT-PCR in Caco-2, HCT116, HT29 cell lines. HDMEC and universal cDNA were used as positive or negative controls respectively.

The mRNA expression of VEGF isoforms, PDGF, HGF and their receptors were determined in three colon cancer cell lines (HCT116, HT29 and Caco-2) by RT-PCR. HDMECs (human dermal microvascular endothelial cell) and universal cDNA were used as positive or negative controls (Figure 1). All cell lines expressed VEGFA and VEGFB but not VEGFC. VEGFR1 was undetectable, whereas VEGFR2 and VEGFR3 showed a weak or negative expression in the cell lines. NRP-1 was expressed equally in all tested cell lines; however, NRP-2 expression varied between lines: it was expressed strongly in Caco-2, weakly in HC116 and not expressed in HT29 cells. The NRP-1 co-receptor HGFR was strongly and equally expressed in all lines, but its principal ligand (HGF) was not expressed. Contrastingly both isoforms of the PDGF ligand, but neither of its receptors were expressed.

### Butyrate downregulates NRP-1 at the mRNA and protein levels

**Figure 2:**
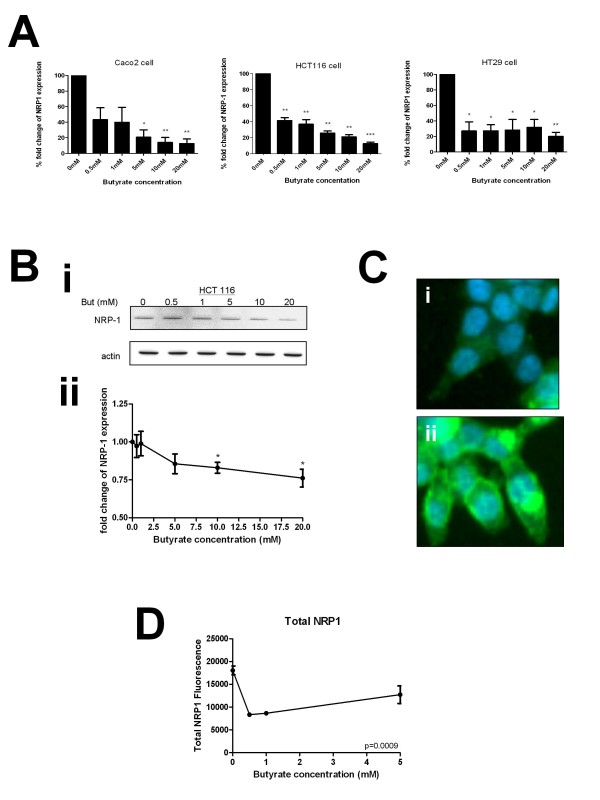
**Butyrate down-regulates NRP-1 at the mRNA and protein level**. HCT116, HT29 and Caco-2 cell lines were treated for 20 hours with media supplemented with increasing concentrations of butyrate. (A) NRP-1 mRNA expression in all three cell lines is significantly reduced when treated with butyrate compared to untreated cells. (B) The level of NRP-1 protein was measured in samples from cells treated with the same range of butyrate concentrations (Bi) shows typical immunoblot for NRP-1 and actin loading control. Intensity was quantitated, normalized to actin and expressed as a proportion of the untreated control (Bii). Reduction was significant at 10 mM and above. *P < 0.05, **P < 0.01, ***P < 0.001. (C) HCT116 cells were grown in 96 well plates, fixed and stained for NRP-1 following butyrate treatment. Panels I & ii show sample images acquired during HCA of the untreated control sample with distinct distributions of NRP-1; (D) Amount of NRP-1 immunofluorescence in cells was quantified by HCA following treatment with butyrate. Significance of decrease across three experiments was determined by ANOVA with p-values inset in the figures.

Preliminary data from a microarray analysis of genes altered in response to butyrate in the cell lines used indicated that NRP-1 expression was reduced (See Additional file 1: Fig S1). Therefore a concentration range of butyrate treatment was undertaken in three colon cancer cell lines (HCT116, HT29 and Caco-2). These lines represent a range of cancer types: HCT116 reportedly has a wild-type p53 response, but is mismatch repair deficient, whereas both HT29 and Caco-2 are p53 deficient chromosome instable lines, and Caco-2 retains differentiation potential. There was a clear concentration-response effect of incremental treatment with butyrate between 0 and 20 mM at the mRNA level (Figure 2A) in all three cell lines. The alteration was pronounced and statistically significant from 0.5 mM. Immunoblotting in HCT116 cells was therefore undertaken in order to confirm that the reduction in NRP-1 mRNA expression would translate to a change in protein expression (Figure 2B). A decrease in NRP-1 protein expression was noticeable at 5 mM butyrate and significant in three independent repeats above 10 mM. Taken together these data show that HCT116, HT29 or Caco-2 cells exposed to butyrate will down-regulate NRP-1 expression through decreased mRNA production leading to reduction in protein levels.

To validate further, we undertook a high-content analysis (HCA) of NRP-1 in HCT116 cells (Figure 2C &2D). The distribution of NRP-1 was very heterogenous (see also Additional file 1: Fig S2 for larger image) and was variously observed as periplasmic, perinuclear and pan-cytosolic. The levels of NRP-1 in cells were quantified by HCA as total NRP-1 (Figure 2D). Following butyrate treatment a decrease in level of NRP-1 cross-reactivity was seen in the cell as a whole consistent with the findings from immunoblotting and qRT-PCR. The functionality of HCA was used to assess whether subcellular distribution was altered however no profound alterations were observed over and above the downgregulation (see Additional file 1: Fig S3).

### Regulation of NRP-1 by transcription factors Sp1 and Sp3

**Figure 3:**
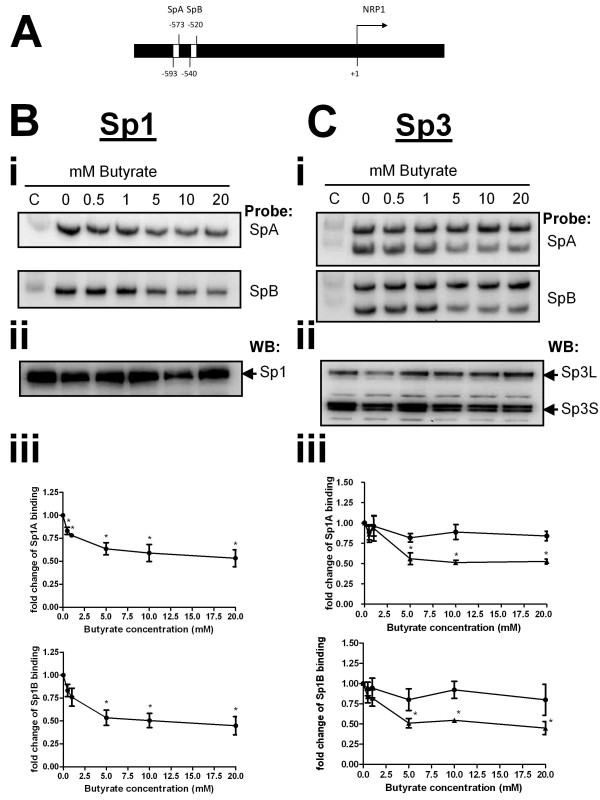
**Sp1 has reduced affinity for the NRP-1 promoter after butyrate treatment**. The organisation of the NRP-1 promoter region, specifically the two distinct Sp consensus sequences SpA and SpB, is shown (A). Nuclear extracts from HCT116 cells treated with 0-20 mM butyrate, were used in a mobility shift assay (WeMSA, previously described [5]), using Sp1antibodies (Panels B) and Sp3 antibodies (Panels C) to determine the binding activities at the two Sp sites. (Bi) Immunoblotting of Sp1 showed that binding activity to SpA and SpB elements was reduced by butyrate in a concentration-dependent manner. (Bii) showed that immunoblotting of Sp1 from nuclear extracts used in binding assays reveals the stable expression of Sp1 before and after treatment with butyrate. (Biii) Densitometry of WeMSA immunoblots indicated that this reduction in binding is significant (*P < 0.05). (C) The same analyses using Sp3 immunoblots revealed that Sp3_S _binding was significantly reduced at both SpA and SpB, whereas Sp3_L _binding remained constant before and after treatment (Panels Cii, Ciii, *P < 0.05).

NRP-1 has previously been shown to be regulated at the transcriptional level by Sp1 and two Sp1 functional consensus sequences occur in the NRP-1 promoter [14]. Our data on butyrate-mediated dysregulation of Bak and p21 suggest alteration in Sp1 binding affinity mediated by increased acetylation of Sp1 is a key event [5]. We therefore investigated the affinity of Sp1 for the NRP-1 promoter following treatments with butyrate known to affect NRP-1 expression. A previously described [5] modified EMSA (WeMSA) was employed to assess alteration in binding of Sp1 to the two putative Sp1 binding sites in the NRP-1 promoter, SpA and SpB (Figure 3A). Increased concentrations of butyrate from 0-20 mM led to decreased affinity of Sp1 for these two target sequences (Figure 3Bi). Nuclear extracts used in binding assays were immunoprobed for Sp1 to confirm that Sp1 levels remained constant before and after treatment with butyrate, indicating that the observed reduction in Sp1 cross-reaction is attributable to reduced DNA-binding affinity of Sp1 (Figure 3Bii). Quantification of three independent repeats of this experiment showed that reduction in binding affinity is progressive and starts at 0.5 mM. The reduction in binding is significant for both target sites following treatment with concentrations of butyrate of 5 mM and above (Figure 3Biii).

In certain cellular contexts and at several genes dysregulated by butyrate, Sp1 appears to be displaced by Sp3 following treatment. We therefore examined the effect of butyrate on binding of both short (S) and long (L) forms of Sp3 at the SpA and SpB binding sites. Both Sp3_L _and Sp3_S _bound to SpA and SpB. Following butyrate treatment binding to both sequences by Sp3_S _was reduced significantly, whereas Sp3_L _binding remained essentially constant (Figure 3Ci). Quantification of three independent repeats showed that the reduction in Sp3_S _binding to both SpA and SpB was significant at 5 mM and above (Figure 3Ciii). Nuclear extracts were also immunoprobed for Sp3 to verify that the observed mobility shift changes were due to altered binding affinity and not attributable to altered expression of Sp3 (Figure 3Cii).

Taken together these data imply that down-regulation of NRP-1 transcription may primarily be through reduced Sp1 and Sp3 binding.

### Conservation of NRP-1 response to HDAC inhibition

**Figure 4:**
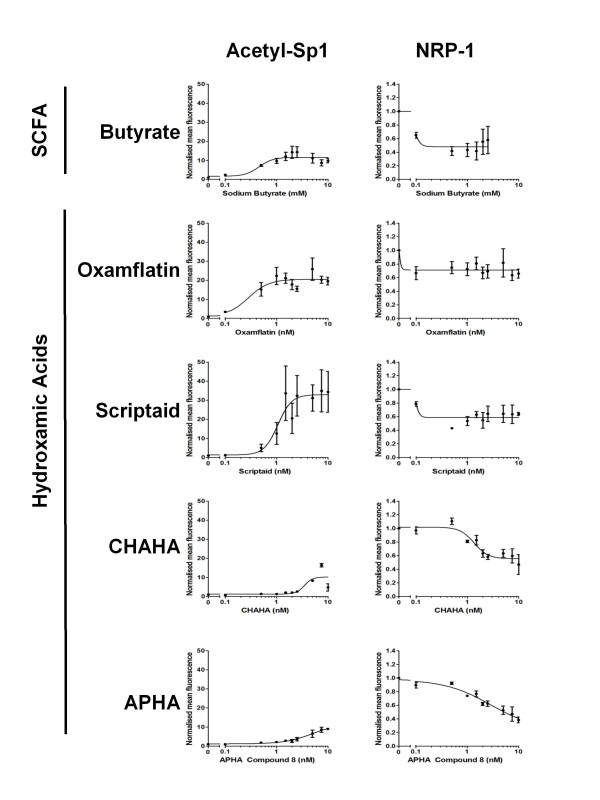
**Analysis of response of NRP-1 to multiple HDAC inhibitors**. The extent of the concomitant response of Sp1 acetylation and NRP-1 down- regulation was determined using a high-content analysis approach. HCT116 cells were treated for 24 hr with concentration ranges of 0-20 mM sodium butyrate, 0-20 μM Oxamflatin, 0-20 μM Scriptaid, 0-20 μM APHA compound 8, 0-20 μM CHAHA. Cells were stained for acetyl-Sp1 and NRP-1 as described in the methods section. Levels of protein are expressed in terms of fluorophore fluorescence relative to that observed in untreated cells, left column shows fluorescence for acetyl-Sp1 and right column for NRP-1.

In order to assess whether the response of NRP-1 expression to butyrate was mediated through butyrate's action as an inhibitor of HDACs, and potentially through acetylation and inhibition of Sp1, we undertook a concentration-response study using a high-content analysis strategy. HCT116 cells were treated with increasing concentrations of butyrate or one of four hydroxamic acids, two of which we have previously shown to induce Sp1 acetylation (oxamflatin and scriptaid), and two with minimal Sp1-acetylating potential (CHAHA and APHA compound 8). Compounds were used over a 2-log concentration range around the known EC_50 _for Sp1 acetylation [6]. Following analysis it was shown that all the HDAC inhibitory compounds reduced NRP-1 expression by around 50% (Fig 4). Butyrate, oxamflatin and scriptaid all induced Sp1 acetylation, but in accordance with our previous study (*ibid*.) the level of Sp1 acetylation following CHAHA and APHA was much lower. Inspection of the concentration-response curves suggested that the EC_50_s for NRP-1 were consistently lower that the EC_50_s for Sp1 acetylation. These data suggest that Sp1 acetylation alone cannot account for the alteration in NRP-1 activity.

### Role of Sp1/3 and HDACs in NRP-1 regulation

**Figure 5:**
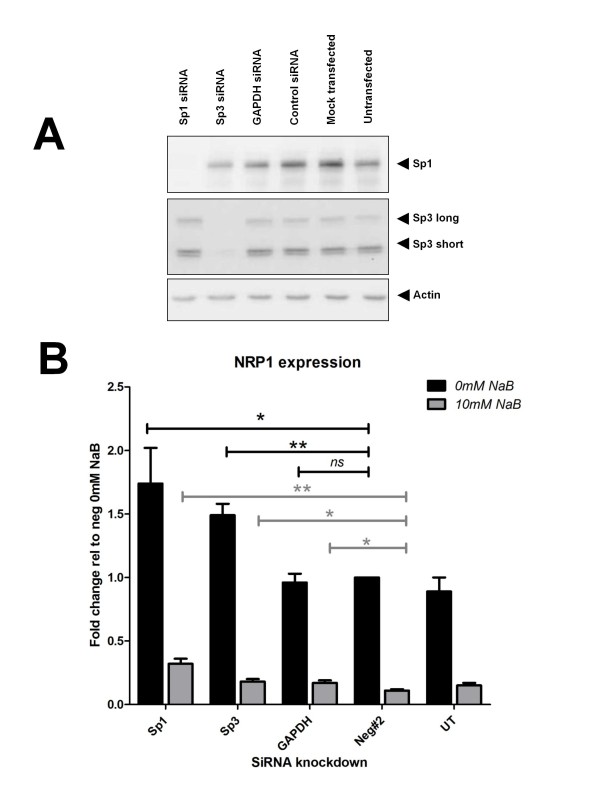
**siRNA reveals the contribution of Sp1, Sp3 and HDACs1, 2, 3 to NRP-1 expression**. Sp1 and Sp3 siRNA were screened for functionality and a single siRNA to each transcription factor was selected for further study. (A) Efficacy and specificity of Sp1 and Sp3 knock-down were shown by immunoblotting protein extracts from transfections and controls 48 hr after transfection. (B) The levels of NRP-1 transcript relative to negative siRNA transfected cells were determined by qRT-PCR using the comparative ΔΔCt method of quantification ns = no significant difference * = P < 0.05, ** = P < 0.01 Student's t-test compared to negative siRNA sample. n = 3 replicates. Error bars represent mean ± s.e

In order further to distinguish the specific roles of Sp1, Sp3 in regulation of Sp1, we undertook a series of siRNA knock-down studies. HCT116 cells were transfected with siRNA to Sp1 and Sp3 and protein lysates analysed by western blot for efficacy and specificity of knock-down (Figure 5A). Reduction in Sp1 and Sp3 levels was substantial, but appeared to cause reduction in cell number (data not shown), perhaps reflecting an essential role of these transcription factors in cell viability. HCT116 cells were transfected with siRNAs against Sp1 and Sp3 (Ambion ID: 143158 and 115336 respectively). 48 hrs post transfection cells were harvested to confirm knockdown at the protein level. Duplicate plates were treated with 0 or 10 mM butyrate for 18 hr. Total RNA was harvested 72 hrs post transfection. Relative NRP-1 expression levels were quantified using Qiagen qRT-PCR gene expression assays to establish the downstream effect of Sp1 or Sp3 knock-down on NRP-1 expression in the presence and absence of butyrate (Figure 5B). Use of a student's t-test demonstrated that knock-down of Sp1 or Sp3 resulted in significant up-regulation of NRP-1 both in the presence and absence of butyrate (P < 0.05). These data are consistent with a model for Sp1 acting as a weak transcriptional activator but with high affinity for DNA, with Sp3 acting as a low-affinity strong transcriptional activator. However the increase in NRP-1 mRNA observed following Sp1 or Sp3 knockdown was not sufficient enough to restore levels to those observed before butyrate treatment. This observation suggests that other acetylation events as a result of butyrate treatment may play a role in butyrate-mediated suppression of NRP-1 mRNA expression.

### Butyrate downregulates VEGF at the mRNA and protein levels

**Figure 6:**
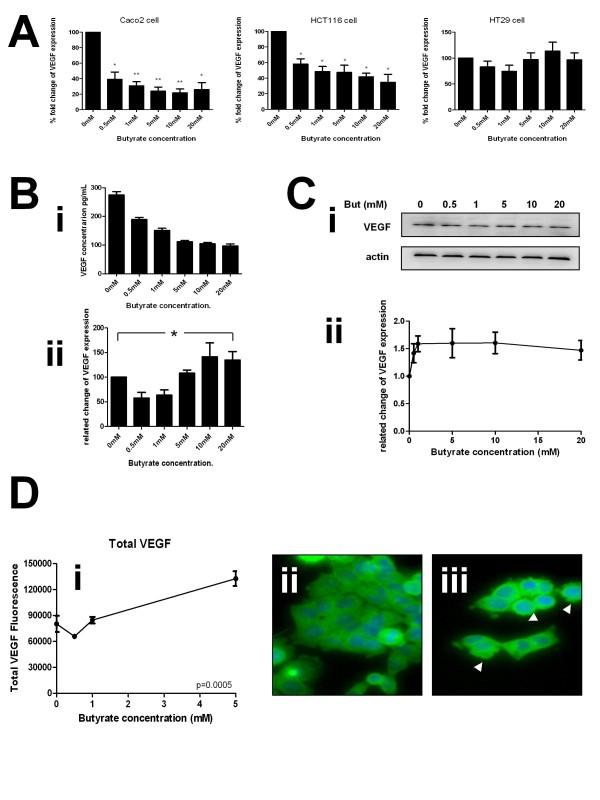
**Butyrate down-regulates VEGF at the mRNA and protein level**. Human colon cancer cell lines were treated with increasing concentrations of butyrate. RNA and protein samples for analysis were described as in fig 2. (A) Butyrate significantly reduced VEGF mRNA expression compared to untreated cells in HCT116 and Caco-2 cell lines whereas there was no significant difference of VEGF expression in HT29 cells. (B) Supernatants of HCT116 cultures treated with increasing concentrations of butyrate were assessed for VEGF levels by ELISA. (Bi) shows the absolute quantitation of VEGF, (Bii) shows the VEGF level normalised to cell count. (C) The level of VEGF protein was established in samples from cells treated with the same range of butyrate concentrations (Ci), shows sample immunoblots of VEGF and actin loading control. Intensity was quantitated, normalized to actin and expressed as a proportion of the untreated control (Cii). *P < 0.05, **P < 0.01. (D) Levels of VEGF in cells and at subcellular locations were determined by HCA. The means ± SD of three independent repeat experiments are shown for total VEGF (subpanel i); perinuclear VEGF (subpanel ii) and cytoplasmic VEGF (subpanel iii). Significance of decrease across three experiments was determined by ANOVA with p-values inset in the figures. Sample HCA images showing distribution of VEGF in untreated cells (subpanel iv) and following treatment with 10 mM butyrate (subpanel v). Butyrate treatment resulted in a more punctuate staining pattern (see arrows).

Butyrate has been shown to affect VEGF expression [16] in certain cellular contexts. To establish the consistency, level and direction of this effect in our panel of cell lines, RNA, protein and supernatant were collected from HCT116, HT29 or Caco-2 cells treated with 0-20 mM butyrate for 20 hours and analyzed by qRT-PCR, immunoblotting or ELISA. In contrast to the results with NRP-1, effects varied by cell line. A pronounced and significant down regulation of VEGF mRNA was seen in the HCT116 and Caco-2 cells, whereas there appeared to be no effect in the HT29 cells (Figure 6A). When the secreted protein levels from HCT116 were measured by ELISA, they were significantly reduced at all tested concentrations of butyrate (Fig 6Bi), although this relationship appears biphasic when corrected for cell mass (Fig 6Bii). This is probably due to the very low numbers of cells at higher concentrations of butyrate treatment causing a denominator effect. In contrast to the mRNA and ELISA data, in the HCT116 cells the total VEGF protein did not decrease in cells, but showed a non-significant trend towards increase (Figure 6C). VEGF levels and subcellular distribution were analysed by HCA as described for NRP-1 (*vide supra*). Subcellular distribution of VEGF was more homogenous in the cell population than NRP-1 (Figure 3Dii). Following treatment with butyrate staining intensity increased with a suggestion of a more punctuate distribution (Figure 3Diii, see arrows). Increase in cellular intensity of VEGF was quantified by HCA and in agreement with data from the western blotting. Data showing VEGF accumulation were highly significant for all events. The functionality of the HCA was used to assess whether redistribution occurred internally for VEGF following butyrate treatment, but no alterations were noted (Additional file 1: Fig S4).

Taken together these data suggest a reduction in VEGF production and signalling by cells following butyrate treatment. We hypothesize that the apparent intracellular increase in VEGF may be due to accumulation owing to cessation of export and synthesis preceding degradation of the protein.

## Discussion

Neuropilin-1 was initially characterised as a receptor for semaphorins which mediate neuronal cell guidance, however, it has more recently been identified as an isoform-specific receptor for VEGF in endothelial cells. Furthermore it is expressed in some tumour cells including colon cancer. While it is known to augment angiogenesis through enhancing the binding of VEGF to VEGF-R2 on endothelial cells, it has also been shown to play an essential role in autocrine anti-apoptotic signalling by VEGF in NRP-1 positive breast cancer cells lacking VEGF-R2 [17,18]. More recently NRP-1 has been shown to be expressed in colon cancer [10] and inhibition of NRP-1 in colon cancer cell lines using siRNA significantly increased cancer cell apoptosis [13]. These data suggest that inhibition of NRP-1 may result in improved prognosis for colon cancer patients, especially as high levels of NRP-1 expression correlated with poor patient survival [13].

Using a panel of tumour-derived lines with epithelial phenotype, we established a profile of gene expression for the families of VEGF ligands and receptors. There was a general consistency in these lines of expression of VEGF A and B, but not C, and little or no expression of any of the classical VEGFR. HGF was not expressed although its receptor was. Contrastingly, the PDGF ligands, but not the receptors were expressed. These data may suggest HGFR as a likely co-receptor with NRP-1, especially as others have shown that this occurs in pancreatic cancer cell lines [19]. However, it is possible that plexins may also be binding partners and these have yet to be studied in non-endothelial cell lines. These data imply expression patterns and roles in epithelia not previously anticipated for NRP-1.

Butyrate is thought to have chemopreventative properties in the colon. We have previously shown that at physiologically relevant concentrations *in vitro *it is a potent inducer of apoptosis in all the cell lines used [5]. We and others have shown that up-regulation of the pro-apoptotic protein Bak occurs in multiple colon cell lines preceding apoptosis and have proposed this as a major contributory mechanism of cell death [5,20,21]. The down-regulation of both VEGF and its receptor NRP-1, shown in this study, suggest an alternative or contributory mechanism to colon cell death following butyrate treatment. The downregulation of NRP-1 is conserved amongst all three cell lines (see fig 2) following butyrate treatment. Critically, there is little or no detectable expression of the principle VEGF receptors in these cell lines, implicating NRP-1, and possibly NRP-2, as the primary VEGF receptor and signal transducer. The down-regulation of NRP-1 is paralleled by the down-regulation of the ligand VEGF, at least at the mRNA level in two of the three cell lines. The regulation of VEGF by butyrate seems more complex as our data suggest that transcription and secretion cease faster than the protein is degraded, leading to an observed increase in intracellular VEGF. This parallels the multi-tier level of regulation previously reported in the study of HIF-regulated genes by butyrate [16]. This may imply a coordinate mechanism of gene regulation, which may have a mechanistic basis [22]. We noted that although there was a clear concentration-responsiveness of downregulation of VEGF by butyrate at the mRNA and secreted protein levels, there was a trend to increased expression at the protein level. We hypothesise that this reflects a cessation of secretion in parallel to downregulation of transcription, but in advance of cessation of translation and protein degradation. The application of a high-content analysis approach was used to study the response of NRP-1 to multiple HDACi. The conservation of NRP-1 downregulation following treatment with multiple HDACi with distinct activities suggests that the downregulation of NRP-1 is mediated through an acetylation-dependent pathway and is not specific to the enterocytic response to butyrate. These data indicate the potential of HDACi as a chemotherapeutic route to suppression of NRP-1 dependent chemoresistance and angiogenesis in the colon.

**Figure 7:**
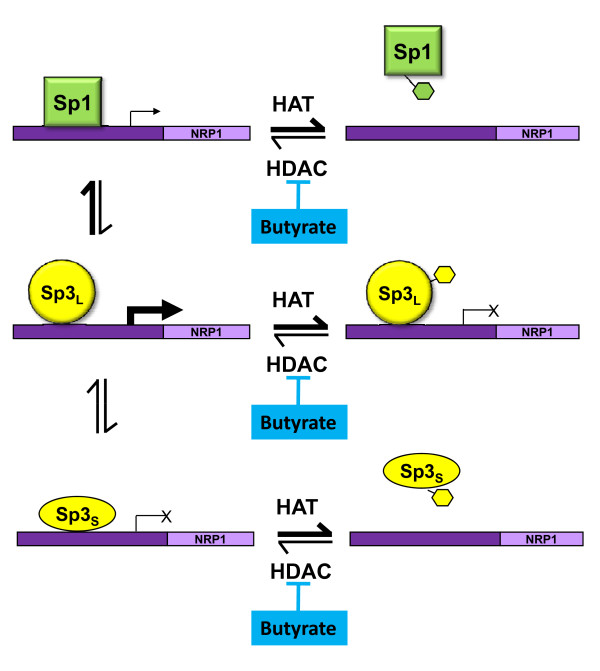
**Model for regulation of NRP-1 expression by Sp transcription factors and HDACs**. The model shows the interaction between Sp1, Sp3_S _and Sp3_L _function in the presence and absence of HDAC inhibition and also accounts for the effects of knockdown of Sp1 or Sp3 using the siRNA strategy.

The downstream signalling pathways of NRP-1 are not yet well understood although progress is being made in the dissection of pathways in endothelial cells [23]. We propose a testable model for the transcriptional regulation of NRP-1 gene, summarized in Fig 7. In this model, the NRP-1 promoter is competed for by Sp1, Sp3_S _and Sp3_L_. There is a conflicted literature around whether Sp3_L _acts as a repressor [24] or transactivator [25,26], although there is more consensus that Sp3_S _is a repressor [26,27]. Our data for Bak suggest that Sp3_L _is a transactivator in this cell line [5]. In our proposed model, in the absence of butyrate, Sp1 has greater affinity for the NRP-1 promoter, but weaker transactivational potential, than Sp3_L_. Sp3_S _acts as a repressor. Following butyrate treatment all the proteins have the potential to become acetylated. We have shown that that acetylation of Sp1 results in reduced DNA binding affinity [6]. The data in Fig 3 suggest that the affinity of both Sp1 and Sp3_S _is reduced following butyrate treatment, which given the constancy of the downregulation in response to other HDACi (see Fig 4), we propose is mediated by acetylation. In contrast Sp3_L _binding is not reduced by acetylation (see fig 3) but is converted to a repressor. This model is also compatible with the data from the siRNA experiment - if Sp1 is knocked down, the transactivational form of Sp3 has access to the promoter, increasing transcription; likewise if Sp3 is knocked down, the repressive action of Sp3_S _is blocked. We anticipate that further studies of this promoter will reveal a more complex model, as this proposal, whilst accommodating all our data, does not yet account for the higher level interaction with other transctivators known at the NRP-1 promoter, including AP-1 [14].

As NRP-1 is involved in both angiogenesis and the prevention of apoptosis in colorectal cancer these data suggest two potential mechanisms for chemoprevention - through the apoptotic regulatory function of NRP-1 and through its pro-angiogenic role. The recent findings that NRP-1 may be expressed in normal epithelia [9,12,23], indicate a role for it in epithelial biology and work must be directed at establishing the extent or otherwise to which endothelial signalling mechanisms are replicated in epithelia.

## Conclusion

Our data therefore suggest routes to improve therapeutic outcome in the colon: through dietary- (or enema-) mediated alteration in luminal butyrate, or through development of specific HDACi to the NRP-1/VEGF pathway. Both of these areas are the subject of our ongoing study.

## Materials and methods

### Colon cancer cell line, cell culture and reagent

Caco-2, HCT116 and HT29 human colon cancer cell lines were kindly donated to Prof C Dive's group (PICR, Manchester, UK). The cells were grown in 1 g/L glucose DMEM (GIBCO) with Penicillin/Streptomycin antibiotics and 10% fetal calf serum (Biosera) at 37°C in 5% CO_2_, 95% air. Dose response effects of sodium butyrate (Calbiochem) were detected by treating cells with culture media supplemented 0 to 20 mM sodium butyrate for 24 hours. 1M stocks of sodium butyrate were made up in PBS and diluted in culture media to give the correct final concentration.

### Protein extraction and immunoblotting

Whole cell lysates were collected by resuspending cell pellets in cell kinase buffer (1M Tris pH5.5, 1M NaF, 1M β-glycerophosphate, 0.2M EDTA, 0.2M EGTA, 10% Triton X-100, 0.1M PMSF, 0.1M NaVO_4 _and 1× proteases inhibitor cocktail, Sigma). Protein concentrations were measured in triplicate by Bradford reagent (Bio-Rad), and 20 μg of protein samples were loaded per well. Transfer membranes were incubated in primary anti-NRP-1 (1:1000 dilution; Santa Cruz) or anti-VEGF (1:1000 dilution; Santa Cruz) for 2 hours at room temperature with gentle agitation, and then in peroxidase-conjugated goat anti-rabbit IgG (1:2000 dilution; Dakocytomation). The immunoblotting results were visualized by CHEMI GENIUS Bio imaging system. The membranes were washed and re-probed with anti-human actin antibody (1:10000 dilution; Abcam) as an equal loading control.

### RNA extraction, RT-PCR and quantitative-PCR (q-PCR)

Total RNA was extracted using Trizol reagent (Invitrogen) following the manufacturer's instructions. Reverse transcription was performed with random primers (Promega) in 1 μg of total RNA using the Superscript III Reverse Transcriptase system (Invitrogen). PCR or qPCR were performed in ReadyMix PCR master mix (Thermo) or 1× SYBR green reagent (QIAGEN) with 1 μl cDNA. Forward and reverse primers for RT-PCR (Sigma-genosys) were designed for amplifying VEGF isoforms and their receptors, NRP-1, NRP-2, PDGFA, PDGFB, PDGFRα, PDGFRβ, HGF, HGFR and actin (Additional file 1: Fig S4). QRT-PCR quantitect assays were purchased from Qiagen for quantitative PCR. QPCR data were collected on the ABI StepOnePlus platform. Relative fold changes were calculated using the ΔΔCT method as previously described [5].

### Westerns of electromobility shift assays (WEMSAs)

An adapted version of the EMSA protocol, a western of a mobility shift gel (WeMSA) was carried out as previously described [5]. Briefly: unlabelled oligonucleotides were incubated with nuclear extracts as per Lightshift EMSA kit instructions; complexes were separated by molecular weight using 5% TBE acrylamide mini-gels in 0.5 × TBE; gels were incubated in SDS buffer (25 mM Tris; 192 mM glycine; 0.2% (w/v) SDS) for 10 min prior to being transferred to PVDF at 100V for 1 hr in 0.5 × TBE; the membranes were blocked in 5% milk TBST for 1 hr prior to immunoprobing and ECL detection of HRP conjugated secondary antibodies. Oligonucleotides for binding assays were commissioned from Sigma Genosys. Oligonucleotides used for for EMSA were 3' biotin-labelled.

### siRNA transfection

siRNA transfections were carried out using Ambion NeoFX as per manufacturer's instructions. Briefly siRNAs and transfection reagent were diluted in OptiMEM (Invitrogen) in two separate tubes. Diluted siRNA and diluted transfection reagent were then mixed together and incubated at room temp for ten min. Complexes were transferred to 24 well plates and 0.5 ml HCT116 cell suspension in antibiotic free media (1 × 10^4 ^cells) were added to each well. A final concentration of 30 nM of siRNA was used. HCT116 cells were transfected with siRNA to Sp1/3 or validated siRNA to HDACs 1, 2 and 3. Controls used were siRNA to GAPDH, a proprietary negative control siRNA, and mock transfected cells. 24 and 48 hours transfection, the cells were collected for RNA and protein extraction respectively, followed by qRT-PCR and immunoblotting analyses.

### Enzyme-linked immunosorbent assay (ELISA)

Conditioned media from cultured cells were collected after each experiment and spun down to remove dead cells and debris. Total VEGF concentration in the culture media was determined by ELISA according to the manufacturer's instruction (R&D system). The degree of intensity was measured using BioTek microplate reader at 450 nm optical density with correction at 560 nm.

### High Content Analysis (HCA)

Cells were grown for HCA analysis as described above, in 96 well plates. Cells were fixed by 3.5% formaldehyde in PBS for 15 minutes at room temperature. NRP-1 (R&D system), acetyl-SP1 (in-house) and VEGF (Abcam) antibodies were diluted in digitonin (500 μl/ml; Sigma) treating cells for 30 minutes at room temperature. The cells were then incubated with anti-sheep Alexa Fluor-blue (Invitrogen) or anti-mouse Alexa Fluor-555 (Invitrogen) antibodies. DNA was stained with Hoechst at 2.5 μg/ml. Plates were analysed on a Cellomics Arrayscan. The Arrayscan compartmental analysis algorithm was used to generate a mask to measure cell surface and cytoplasmic staining independently.

### Statistical analysis

The results were expressed as mean ± standard error of the mean (SEM). Densitometric analysis of western blots was performed by Gene Tool software from SynGene to quantify the results of western blotting and westerns of electromobility shift assays. All statisical analyses were conducted using SPSS v18 software (Chicago, IL, USA) and Prism 5. Statistical significance was determined using one-way ANOVA in ELISA and HCA results, and Student's t test was used in densitometric and qRT-PCR results. Each experiment was repeated at least 3 times. P < 0.05 was considered statistically significant.

## Competing interests

The authors declare that they have no competing interests.

## Authors' contributions

CWY undertook experiments and drafted the manuscript, JSW designed and undertook experiments, HC undertook all pilot experiments, CAS co-conceived and co-directed the work, BMC co-conceived and co-directed the study. All authors read and approved the final manuscript.

## Supplementary Material

Additional file 1 **Supplementary figures**. Fig S1. Pilot data generated from microarray Fig S2. Additional microscopy images supporting data in the paper. Fig S3. Data on the subcellular localisation of NRP-1 and VEGF following butyrate treatment. Fig S4. Detailed description of PCR primers used Click here for file
