# Supplementary material for: Butyrate suppresses expression of neuropilin I in colorectal cell lines through inhibition of Sp1 transactivation
Source: Mol Cancer. 2010 Oct 15;9:276. doi: 10.1186/1476-4598-9-276 (PMC2974727; doi:10.1186/1476-4598-9-276)
Supplement: Additional file 1 — Supplementary figures. Fig S1. Pilot data generated from microarray Fig S2. Additional microscopy images supporting data in the paper. Fig S3. Data on the subcellular localisation of NRP-1 and VEGF following butyrate treatment. Fig S4. Detailed description of PCR primers used [file 1476-4598-9-276-S1.ppt]

## Slide 1
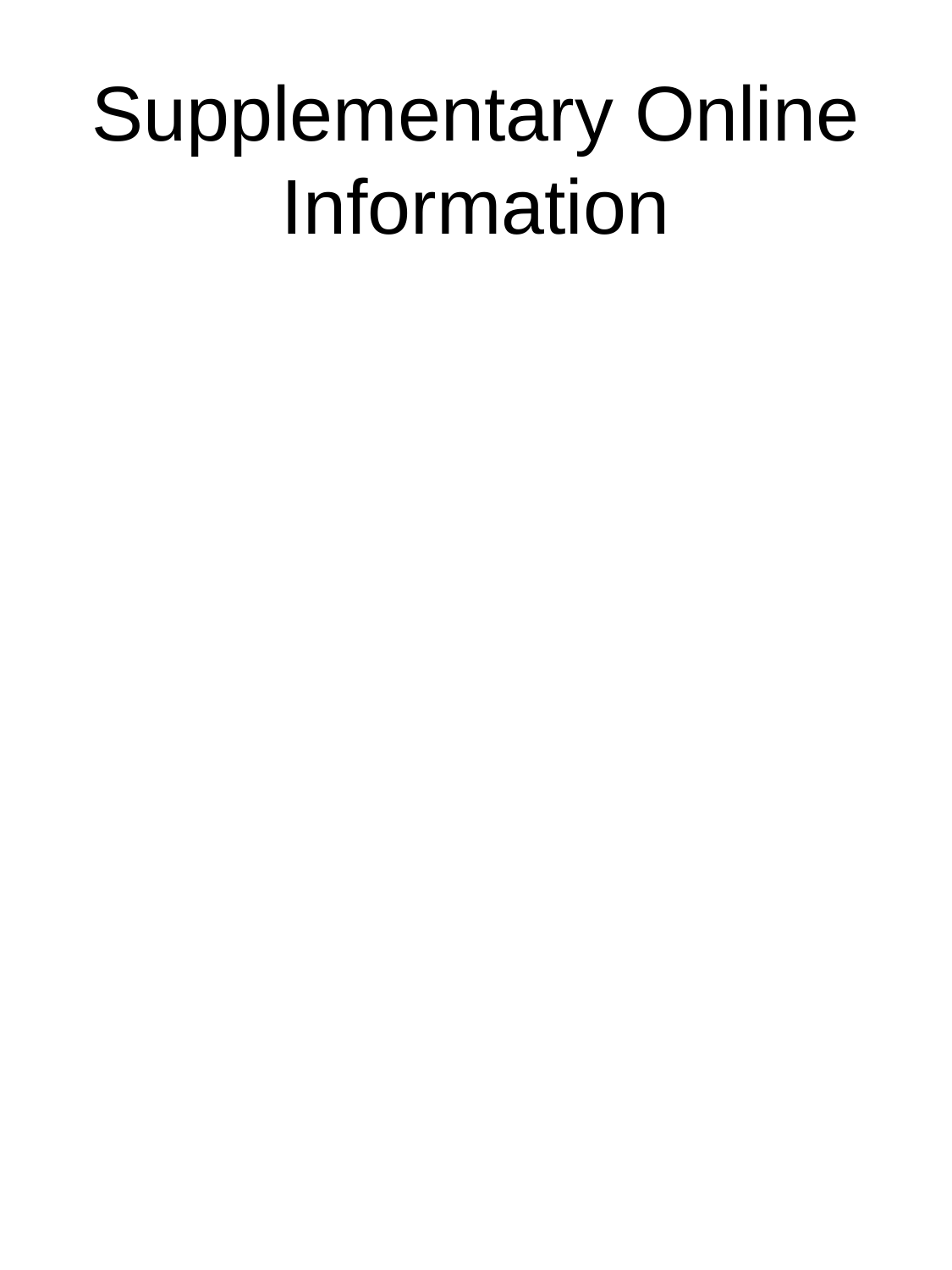

# Supplementary Online Information

## Slide 2
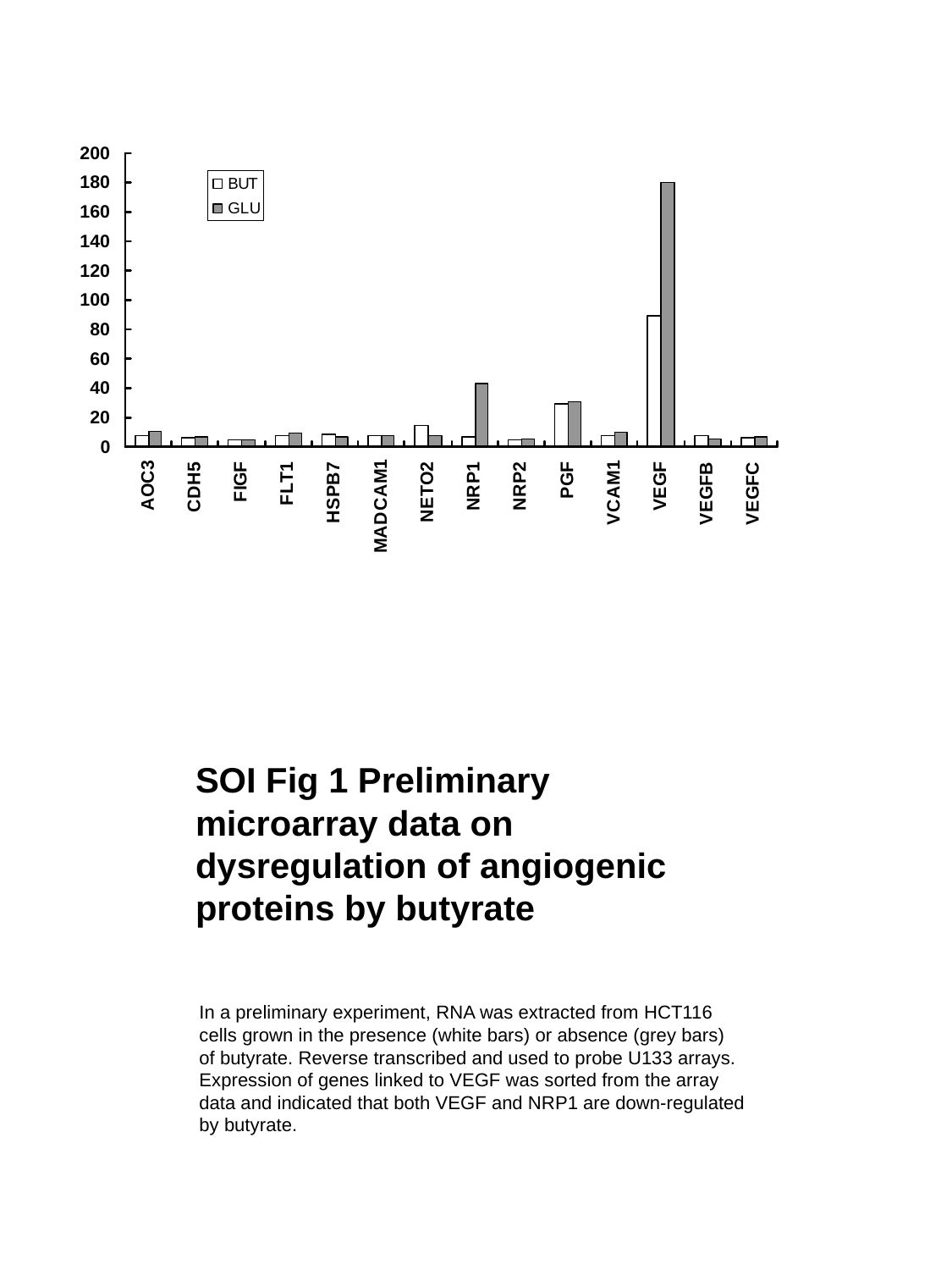

# SOI Fig 1 Preliminary microarray data on dysregulation of angiogenic proteins by butyrate
In a preliminary experiment, RNA was extracted from HCT116 cells grown in the presence (white bars) or absence (grey bars) of butyrate. Reverse transcribed and used to probe U133 arrays. Expression of genes linked to VEGF was sorted from the array data and indicated that both VEGF and NRP1 are down-regulated by butyrate.

## Slide 3
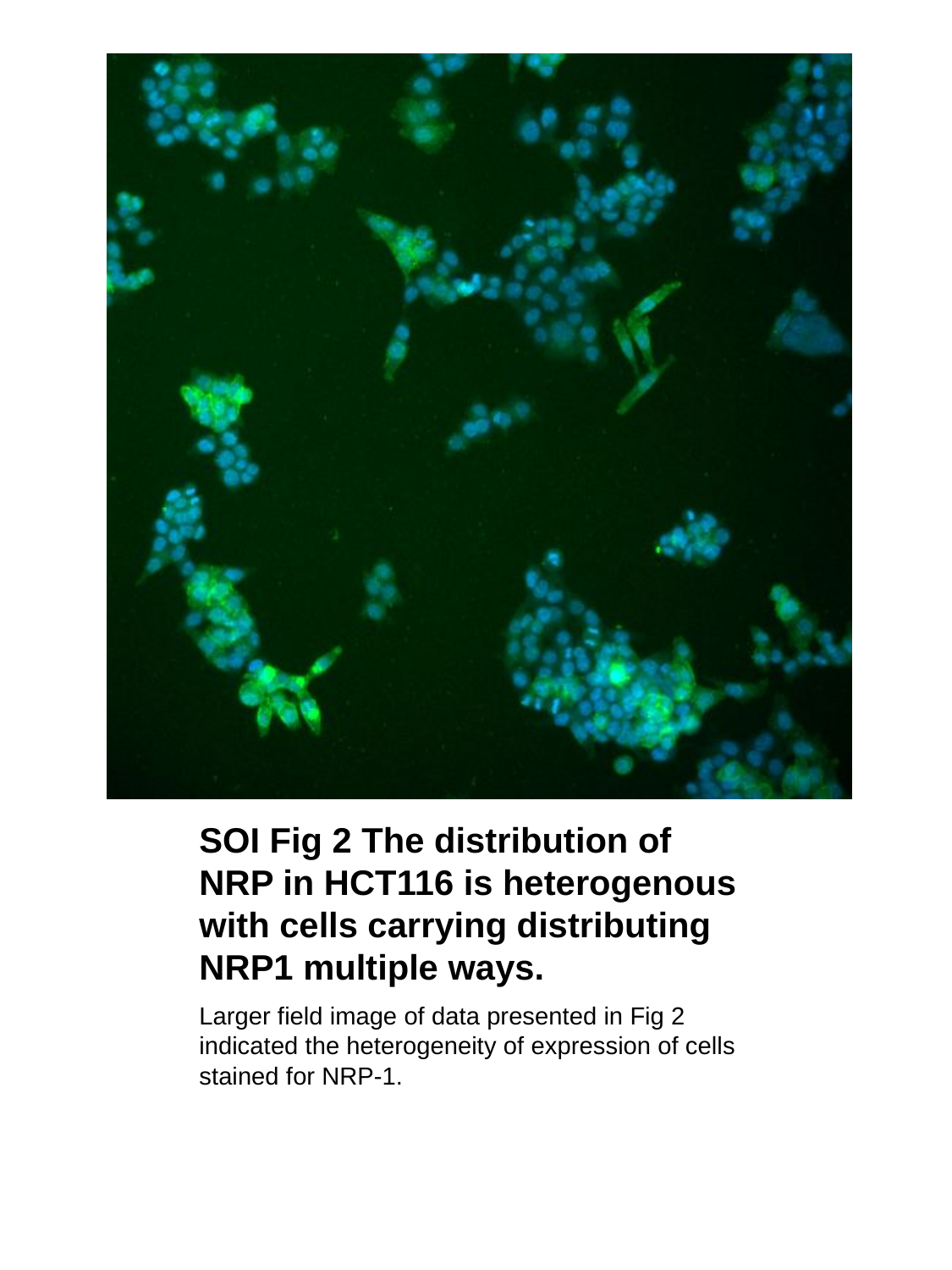

# SOI Fig 2 The distribution of NRP in HCT116 is heterogenous with cells carrying distributing NRP1 multiple ways.
Larger field image of data presented in Fig 2 indicated the heterogeneity of expression of cells stained for NRP-1.

## Slide 4
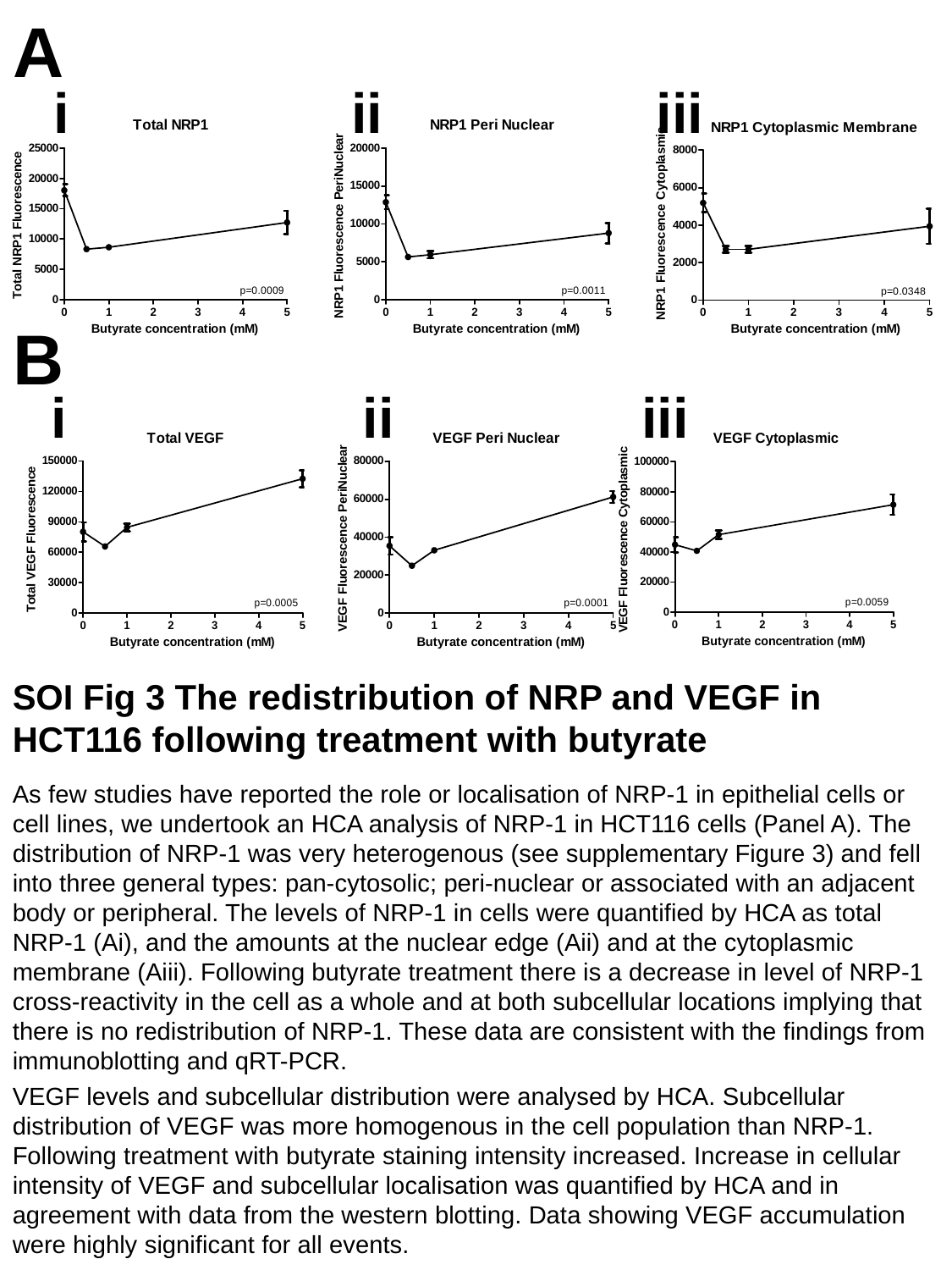

A
i
ii
iii
B
i
ii
iii
SOI Fig 3 The redistribution of NRP and VEGF in HCT116 following treatment with butyrate
As few studies have reported the role or localisation of NRP-1 in epithelial cells or cell lines, we undertook an HCA analysis of NRP-1 in HCT116 cells (Panel A). The distribution of NRP-1 was very heterogenous (see supplementary Figure 3) and fell into three general types: pan-cytosolic; peri-nuclear or associated with an adjacent body or peripheral. The levels of NRP-1 in cells were quantified by HCA as total NRP-1 (Ai), and the amounts at the nuclear edge (Aii) and at the cytoplasmic membrane (Aiii). Following butyrate treatment there is a decrease in level of NRP-1 cross-reactivity in the cell as a whole and at both subcellular locations implying that there is no redistribution of NRP-1. These data are consistent with the findings from immunoblotting and qRT-PCR.
VEGF levels and subcellular distribution were analysed by HCA. Subcellular distribution of VEGF was more homogenous in the cell population than NRP-1. Following treatment with butyrate staining intensity increased. Increase in cellular intensity of VEGF and subcellular localisation was quantified by HCA and in agreement with data from the western blotting. Data showing VEGF accumulation were highly significant for all events.

## Slide 5
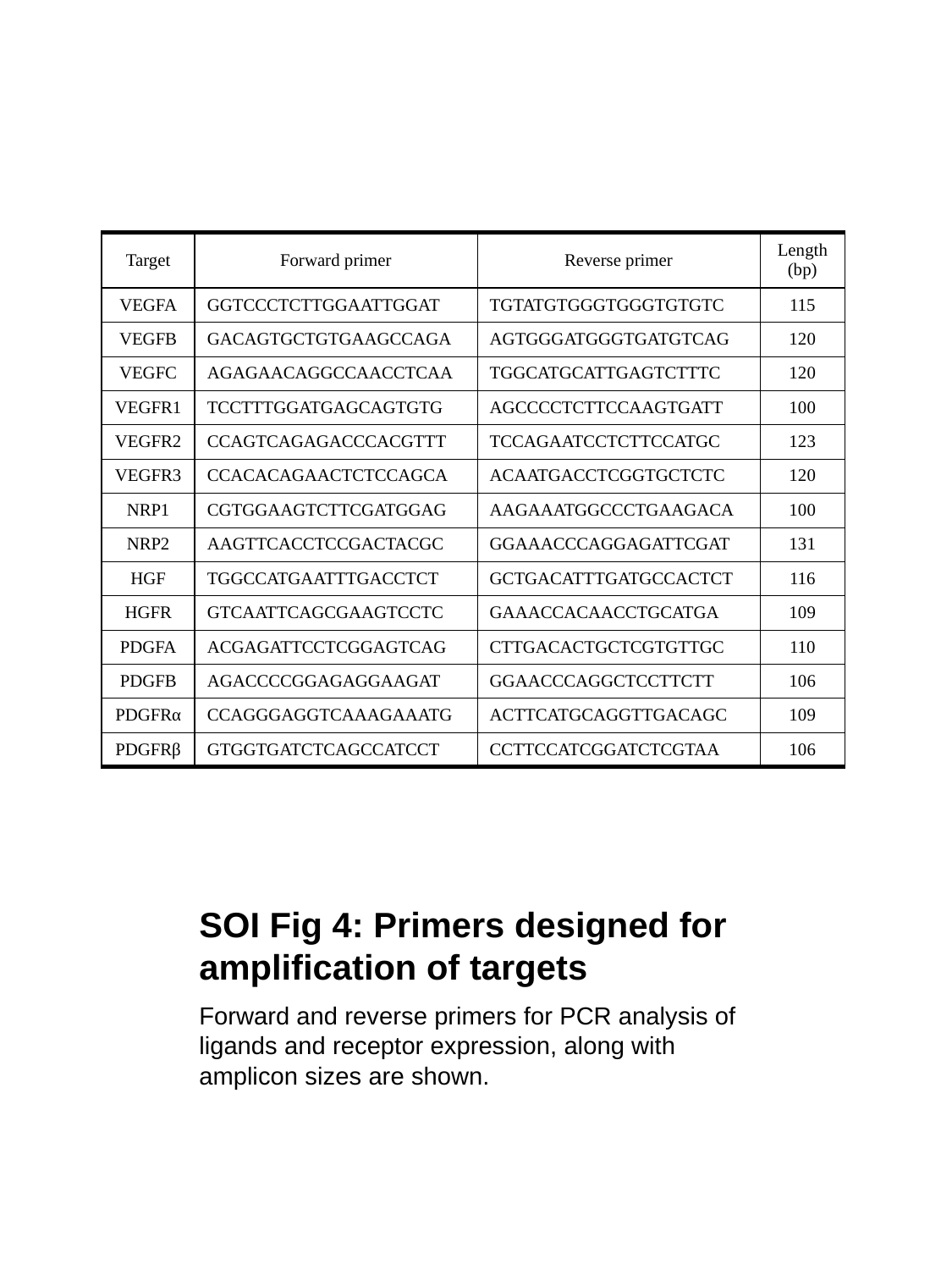

| Target | Forward primer | Reverse primer | Length (bp) |
| --- | --- | --- | --- |
| VEGFA | GGTCCCTCTTGGAATTGGAT | TGTATGTGGGTGGGTGTGTC | 115 |
| VEGFB | GACAGTGCTGTGAAGCCAGA | AGTGGGATGGGTGATGTCAG | 120 |
| VEGFC | AGAGAACAGGCCAACCTCAA | TGGCATGCATTGAGTCTTTC | 120 |
| VEGFR1 | TCCTTTGGATGAGCAGTGTG | AGCCCCTCTTCCAAGTGATT | 100 |
| VEGFR2 | CCAGTCAGAGACCCACGTTT | TCCAGAATCCTCTTCCATGC | 123 |
| VEGFR3 | CCACACAGAACTCTCCAGCA | ACAATGACCTCGGTGCTCTC | 120 |
| NRP1 | CGTGGAAGTCTTCGATGGAG | AAGAAATGGCCCTGAAGACA | 100 |
| NRP2 | AAGTTCACCTCCGACTACGC | GGAAACCCAGGAGATTCGAT | 131 |
| HGF | TGGCCATGAATTTGACCTCT | GCTGACATTTGATGCCACTCT | 116 |
| HGFR | GTCAATTCAGCGAAGTCCTC | GAAACCACAACCTGCATGA | 109 |
| PDGFA | ACGAGATTCCTCGGAGTCAG | CTTGACACTGCTCGTGTTGC | 110 |
| PDGFB | AGACCCCGGAGAGGAAGAT | GGAACCCAGGCTCCTTCTT | 106 |
| PDGFRα | CCAGGGAGGTCAAAGAAATG | ACTTCATGCAGGTTGACAGC | 109 |
| PDGFRβ | GTGGTGATCTCAGCCATCCT | CCTTCCATCGGATCTCGTAA | 106 |
# SOI Fig 4: Primers designed for amplification of targets
Forward and reverse primers for PCR analysis of ligands and receptor expression, along with amplicon sizes are shown.
